# Risk Assessment Acknowledging Variability in Both Exposure and Effect

**DOI:** 10.1021/acs.est.2c06088

**Published:** 2022-10-07

**Authors:** Nico M. van Straalen, Klaas H. den Haan, Joop L. M. Hermens, Kees van Leeuwen, Dik van de Meent, John R. Parsons, Pim de Voogt, Dick de Zwart

**Affiliations:** † A-LIFE Ecology and Evolution, Vrije Universiteit Amsterdam, De Boelelaan 1087, 1081 HV Amsterdam, The Netherlands; ‡ ARES, Association of Retired Environmental Scientists, Maria van Boechoutlaan 25, 3984 PE Odijk, The Netherlands

**Keywords:** chemicals policy, REACH, risk assessment, predicted environmental concentration, fate modeling, no-effect concentration, risk quotient, exposure, toxicity, species sensitivity

Risk assessment provides a scientific basis for evaluating potentially toxic chemicals. Central in the concept of risk is its dependence on both exposure and toxicity. Chemicals will only express their toxicity when these exceed a concentration at which a defined target becomes affected. While this is a trivial remark among environmental risk assessors, the new chemicals policy of the EU tends to divert into a different direction. The communication of the European Commission on its new Chemicals Policy places an emphasis on hazard-based chemical management.^1^ We urge the Commission to reconsider this view. We believe that for the majority of chemicals evaluated under REACH, a scientifically sound risk assessment evaluating both exposure and effect should remain the core of the EU strategy “towards a toxic-free environment”.

Environmental risk assessment originated in the very beginning of environmental science, in the 1960s, when environmental chemists discovered the global distribution of persistent compounds in the environment and wildlife. Thereafter, chemists and biologists, in a joint effort, have developed a strategy for phasing out toxics, producing new, safer chemicals, and mitigating polluted sediments and soils. In the course of the 1980s, risk assessment of chemicals was solidified as a broadly accepted principle. At its basis is a comparison of estimated exposure concentrations with estimated effect concentrations. This often takes the form of a simple ratio applicable to each individual chemical: the predicted environmental concentration relative to the no-effect concentration (PEC/NEC). A ratio of >1 triggers action and usually prohibits approval, according to guidelines of the REACH program. Later, further developments in risk assessment science have led to the notion that this simple quotient approach suffered from a number of serious shortcomings, the most important being that simple PEC/PNEC quotients do not adequately measure the extent of ecological risks of chemicals.^2^ The reason is that both PEC and NEC are stochastic variables that follow probability distributions. In the case of NEC, this was implemented in the “species sensitivity distributions” approach;^3^ however, for PEC, a single exposure concentration (ignoring the possibility of having smaller or greater values) is commonly assumed in risk assessment.

The simple quotient approach has become the basis of environmental risk assessment of chemicals throughout the world. We argue, however, that the ambition of REACH (i.e., safe use of chemicals) is supported best by considering the entire distribution of exposure and effect concentrations. This yields a quantitative measure for the probability that exposure concentrations exceed critical effect concentrations.^4^ This measure is called “expected risk”. The “expected risk” approach was developed originally as a statistical method for quantifying the so-called probability of failure. We argue that the “expected risk” procedure, rather than the simple PEC/PNEC quotient approach, provides the better basis for risk assessment. The following arguments apply.

- The simple risk quotient (RQ) method merely tests whether PEC is lower than NEC, without stating the extent to which this is the case, while the expected risk calculation procedure (ER) is a mathematically proven procedure for quantifying the probability that the exposure concentration PEC exceeds the critical effect concentration NEC.
- In the ER approach, chemicals can be compared or ranked according to the value of ER, whereas RQ offers no scientific rationale for doing so.
- ER easily allows extensions to deal with mixtures of chemicals (by propagation of probabilities), while RQ can serve this such purpose only for mixtures of chemicals that share the same toxicological mode of action.
- In REACH, the “possibility to use a chemical safely” is evaluated by testing whether RQ is <1, using the simple RQ method. A positive outcome of this evaluation is that the risk of using the tested chemical is smaller than the maximum acceptability level of 0.05 (5%). The European Commission should be aware that a positive outcome should not be interpreted as “chemical safety”. Such an outcome could be obtained from using the integrated ER method, which would make it possible to test whether the risk is acceptably low. If ER falls below the widely adopted negligibility level of 10^–6^, it would be reasonable to conclude from the test that a the chemical “can be used safely”.
- By ignoring uncertainty and variability of exposure concentrations, RQ systematically underestimates the probability that exposure concentrations exceed critical effect concentrations. ER does not ignore the stochastic nature of PEC but accounts for it in the value of ER. In contrast to RQ, ER is not uncertain, but merely greater the more unprecise PEC and NEC are.

The methodology for estimating “expected risk” from emission scenarios and species sensitivity distributions is readily available and validated.^3,4^ We, an association of retired scientists with due experience in risk assessment, call upon the European Commission to move forward in implementing modern, high-throughput approaches for risk assessment, which, compared to simple hazard management, include much more precision and allow a better weighting of costs and benefits associated with the use of synthetic chemicals in modern society.
